# CRISPR for companion diagnostics in low-resource settings†

**DOI:** 10.1039/d4lc00340c

**Published:** 2024-09-05

**Authors:** Xu Qian, Qiang Xu, Christopher J. Lyon, Tony Y. Hu

**Affiliations:** a Department of Clinical Laboratory, Zhejiang Cancer Hospital, Hangzhou Institute of Medicine (HIM), Chinese Academy of Sciences Hangzhou 310022 China Qian_michelle2014@163.com; b Center for Cellular and Molecular Diagnostics, Tulane University School of Medicine 1430 Tulane Ave New Orleans LA 70112 USA tonyhu@tulane.edu; c Department of Biochemistry and Molecular Biology, Tulane University School of Medicine 1430 Tulane Ave New Orleans LA 70112 USA

## Abstract

New point-of-care tests (POCTs), which are especially useful in low-resource settings, are needed to expand screening capacity for diseases that cause significant mortality: tuberculosis, multiple cancers, and emerging infectious diseases. Recently, clustered regularly interspaced short palindromic repeats (CRISPR)-based diagnostic (CRISPR-Dx) assays have emerged as powerful and versatile alternatives to traditional nucleic acid tests, revealing a strong potential to meet this need for new POCTs. In this review, we discuss CRISPR-Dx assay techniques that have been or could be applied to develop POCTs, including techniques for sample processing, target amplification, multiplex assay design, and signal readout. This review also describes current and potential applications for POCTs in disease diagnosis and includes future opportunities and challenges for such tests. These tests need to advance beyond initial assay development efforts to broadly meet criteria for use in low-resource settings.

## Abstract 2

New point-of-care tests (POCTs), which are especially useful in low-resource settings, are needed to expand screening capacity for diseases that cause significant mortality: tuberculosis, multiple cancers, and emerging infectious diseases.

## Graphical abstract

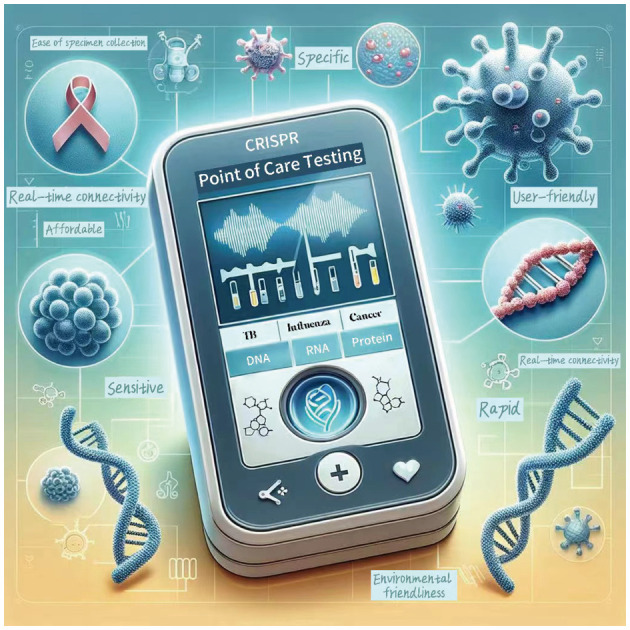

## 1 Introduction

Rapid and accurate clinical tests are essential for effective patient care in multiple disease conditions, as they can provide critical information for disease diagnosis, treatment planning, and prognosis. Such tests can also play essential roles in efforts to contain various infectious diseases, as was demonstrated during the COVID-19 pandemic, when multiple tests for SARS-CoV-2 were rapidly developed and employed in screening efforts intended to reduce virus transmission. But access to sensitive and accurate diagnostic tests is often limited in many parts of the world regarded as low-resource settings. These tests often require expensive equipment and well-trained personnel and are thus frequently restricted to central laboratories that have the resources and infrastructure required to perform them. Self-contained point-of-care tests (POCTs), which do not require extensive training or additional resources to perform, have the potential to extend the use of critical diagnostic/prognostic assays into low-resource settings to improve healthcare disparities and patient outcomes and inform disease control strategies for future outbreaks.

Ideal POCTs should adhere to the ASSURED criteria (affordable, sensitive, specific, user-friendly, rapid, equipment-free, delivered),^1^ and it has recently been proposed that these should be expanded to the REASSURED criteria, including real-time connectivity, ease of specimen collection, and environmental friendliness.^2^ Sensitivity and specificity should largely be maintained in REASSURED assays for low-resource settings, but performance may drop slightly compared with that of central laboratory tests to allow for accessibility and affordability in these settings. Tremendous progress has been made in the development and adoption of assays that meet ASSURED criteria; these assays include diagnostic tests for HIV-1, malaria, and syphilis.^2^ But major challenges remain for many other diseases, particularly for those requiring nucleic acid (NA)-based assays, which may need careful refinement to ensure acceptable performance after exposure to a variety of conditions during assay shipping, storage, and use.

**Fig. 1:**
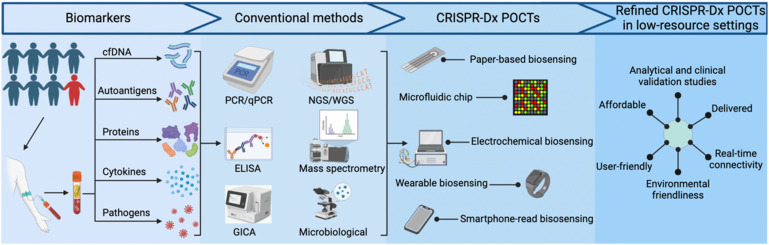
Development of new CRISPR-based assays for point-of-care diagnostics. Such diagnostic systems should incorporate REASSURED criteria (real-time connectivity, ease of specimen collection, affordable, sensitive, specific, user-friendly, rapid, equipment-free, deliverable to end-users, and environmentally friendly). cfDNA: cell free DNA; GICA: colloidal gold immunochromatography assay; PCR: polymerase chain reaction; ELISA: enzyme-linked immunosorbent assay; WGS: whole genome sequencing; NGS: next generation sequencing.

The rapid expansion of available genomic data over the past two decades serves as an excellent resource for new NA tests designed to identify specific microbial pathogens, predict drug susceptibility or disease progression, or provide other useful clinical information. Such NA tests can have excellent diagnostic or prognostic value, but require highly-trained personnel, careful sample preparation, and costly equipment and/or reagents that limit their utility. Adopting various isothermal amplification approaches can alleviate the need for thermocyclers, required by conventional PCR assays, but these alternative approaches often have their own distinct limitations. Clustered regularly interspaced short palindromic repeats (CRISPR)-based diagnostic (CRISPR-Dx) assays, such as SHERLOCK,^3^ DETECTR,^4^ and HOLMES,^5^ have emerged as powerful and versatile tools that complement traditional NA assays for sensitive detection of specific NA sequences in complex samples. Other target types, including proteins and small molecules, can also be detected by CRISPR-based methods that employ aptamers or proteins that recognize these targets. CRISPR-Dx assays thus exhibit robust potential for adaptation to POCTs, as has been reported in studies that have used paper-based,^6^ microfluidic chip,^7,8^ electrochemical,^9^ wearable^10^ and smartphone-read biosensor^11^ approaches that can be refined for use in low-resource settings (Fig. 1).^7,8,12–16^ Importantly, similar to the development of clinical assays, the development of refined CRISPR-Dx POCTs requires analytical and clinical validation. These tests should also meet REASSURED criteria.

This review describes how the inherent features of CRISPR-based assays align with the needs of POCTs and highlights progress made on CRISPR-Dx applications for infectious disease. We also discuss new applications of CRISPR-based assays in oncology, including their broad potential for cancer prevention, screening, and chronic disease management.

Finally, we discuss barriers remaining for the broad adoption of CRISPR-based applications, their utility in low-resource settings, and the future outlook for these assays.

## 2 CRISPR-Dx assays

### 2.1 Mechanism of CRISPR/Cas-based diagnosis

**Fig. 2:**
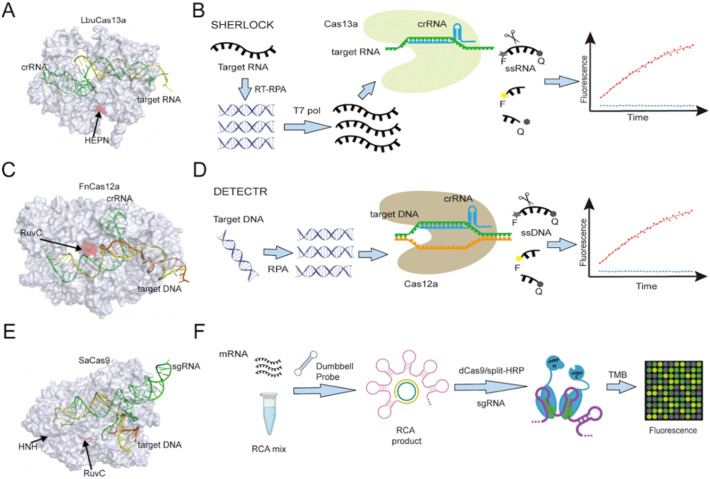
(A) Structure of Cas13a (PDB ID: 5XWP) protein complexed with crRNA and bound to a target NA sequence recognized by the crRNA sequence. (B) Cas13-based SHERLOCK assay workflow scheme. (C) Structure of Cas12a (PDB ID: 6GTG) protein with crRNA and a target NA sequence. (D) Cas12a-based DETECTR assay workflow scheme. (E) Cas9 (PDB ID: 5AXW) with crRNA and a target NA sequence. (F) A dCas9-based platform utilizes split horseradish peroxidase for detection of microRNA. RuvC domain catalytic sites are marked in Cas12a and Cas9 protein, as is the HEPN domain catalytic site in Cas13a and the HNH domain catalytic site in Cas9. RCA: rolling circle amplification; TMB: 3,3′,5,5′-tetramethylbenzidine.

CRISPR/CRISPR-associated protein (CRISPR/Cas) complexes function as adaptive immune system factors in bacteria and archaea by targeting foreign NAs for destruction.^17^ Each CRISPR/Cas complex employs a short (17 to 20 nucleotide) CRISPR RNA sequence (crRNA) transcribed from a CRISPR array to recognize and directly cleave previously encountered pathogen-specific NA target sequences (Fig. 2), which may or may not contain a conserved protospacer adjacent motif (PAMs) at its 3′ end, depending upon the CRISPR/Cas system. The potential of these Cas/crRNA complexes to cleave a diverse array of NA target sequences was rapidly recognized and CRISPR/Cas systems have since become the gold-standard approach for genome editing. After binding their recognition sequence, some Cas protein types exhibit both target-specific (*cis*), and non-specific collateral (*trans*) cleavage activities. Such *trans*-cleavage activities can indiscriminately cleave single-stranded DNA (*e.g.*, Cas12 (ref. 4 and 18) or RNA (*e.g.*, Cas13 (ref. 19)), depending on the Cas effector, and have thus been employed as an efficient means of cleaving a labelled reporter probe or other substrates that can induce a detectable assay signal.

The most commonly used Cas systems for CRISPR-Dx assays are Cas13, Cas12, Cas9, and Cas14. These are all class 2 CRISPR–Cas systems that employ a single Cas effector protein. Within class 2, Cas9 is classified as type II; Cas12 and Cas14, as type V; and Cas13, as type VI. These Cas systems have distinct features, including different crRNA structures, PAM sequences, target specificities, and cleavage activities, which give them specific utility for applications that detect various NA targets.

#### 2.1.1 Cas13

Cas13 contains two higher eukaryotes and prokaryotes nucleotide-binding (HEPN) domains that mediate the *cis*- and *trans*-cleavage activity observed when a single-stranded RNA target sequence is recognized by the crRNA of a CRISPR/Cas13 complex (Fig. 2A).^19,20^ CRISPR/Cas13 complexes bound to a target RNA sequence exhibit high *trans*-cleavage activity and can produce 10^4^ non-specific *trans*-cleavage events per every target-specific *cis*-cleavage event,^20^ and thus most Cas13-based molecular diagnostics use this collateral cleavage activity to amplify the detection of their specific RNA targets.

The SHERLOCK (specific high-sensitivity enzymatic reporter unLOCKing) platform was the first reported assay based on Cas13 collateral RNase activity (Fig. 2B).^3,21,22^ This assay amplifies its targeted DNA or RNA of interest by recombinase polymerase amplification (RPA) or reverse transcription (RT) plus RPA. It then employs T7 RNA polymerase to transcribe its amplicons into RNA that can be recognized by a target-specific Cas13/crRNA complex to trigger Cas13-mediated *trans*-cleavage of a reporter RNA with both a fluorophore and a quencher. The fluorescent signal produced by this process can be monitored in real time, enabling attomolar detection of a target RNA, which is comparable to the detection achieved by digital droplet PCR (ddPCR) or quantitative PCR (qPCR). SHERLOCK was tested for its ability to detect Zika and Dengue viruses in clinical isolates, genotype bacteria, and detect single-nucleotide polymorphisms (SNPs). In the latter, it could detect low-frequency cancer-associated mutations (EGFR L858R and BRAF V600E) present in cell-free DNA (cfDNA) fragments spiked into mock patient samples. Several modifications have been made to the original sample processing, target amplification, and signal readout methods of the SHERLOCK platform to adapt it for new applications and make the platform more accessible to its end users, some of these modifications are discussed in later sections.

Efforts have also been made to modify Cas13 activity, including a recent study that demonstrated that Cas13 collateral cleavage activity could be enhanced by tethering its RNA binding domain adjacent to the active site responsible for these *trans*-cleavage events. These engineered Cas13 variants can exhibit catalytic efficiencies for reporter cleavage that are two- to three-fold higher than wildtype Cas13.^23^ This study suggests the potential for protein engineering to further improve detection sensitivities beyond what's currently achievable with Cas13-based assays and suggests that similar optimization efforts could possibly also improve the detection sensitivities of assays based on other Cas protein systems.

#### 2.1.2 Cas12

Cas12a/crRNA complexes have specific *cis*-cleavage activity when bound to single- or double-stranded DNA (ssDNA or dsDNA) complementary to their crRNAs but also exhibit robust non-specific *trans*-cleavage activity for single-stranded DNA that interacts with their collateral cleavage site (Fig. 2C).^4,18^ This property was first employed to generate the DNA endonuclease targeted CRISPR *trans* reporter (DETECTR) assay that used RPA to amplify a human papillomavirus (HPV) target region detected by Cas12a cleavage of a quenched fluorophore-labelled dsDNA reporter probe (Fig. 2D).^4^ This assay exhibited attomolar sensitivity for samples spiked with plasmids containing the targeted HPV region and detected HPV in more complex biological samples, including cultured cells and patient anal swab samples. A similar, independently developed system named HOLMES (a one-hour low-cost multipurpose highly efficient system) was reported to detect pseudorabies virus or Japanese encephalitis virus with a sensitivity of 1 to 10 attomolar.^5^ The initial version of HOLMES was based on Cas12a and used PCR or RT-PCR for target amplification, but a subsequent version of this assay platform (HOLMESv2)^24^ was developed to employ thermophilic Cas12b and use loop-mediated isothermal amplification (LAMP) for amplification.

#### 2.1.3 Cas9

Cas9/crRNA complexes preferentially recognize and cleave dsDNA targets, but these complexes lack *trans*-cleavage activity, and their use in diagnostic applications thus relies solely on their precise recognition of a target dsDNA sequence (Fig. 2E). Cas9 mutants that are catalytically dead (dCas9) or that are only able to produce single-stand nicks (nCas9) are thus often employed in Cas9-based diagnostics to prevent the destruction of their dsDNA targets and maximize the signal from the bound Cas9/crRNA complex, which can be generated by a variety of approaches. For example, one research group generated a dCas9 system where two dCas9 proteins were fused with either the N- or C-terminal domains of firefly luciferase, and adjacent binding of both dCas9 fusion proteins on a DNA target led to the stable interaction of the two luciferase domains, producing a signal.^25^ In a similar approach, another group generated a dCas9 assay system where dCas9 proteins were fused with the N- or C-terminal regions of horseradish peroxidase (Fig. 2F).^26^ The group also combined this dCas9 assay approach with a rolling cycle amplification (RCA) reaction to convert the microRNA (miRNA) target to dsDNA and amplify its abundance. Such dCas9 dimerization assays can increase specificity but may also reduce assay kinetics or target sensitivity. To improve sensitivity in these assays, some researchers have tried assessingdCas9 binding to a target DNA sequence by changes in nanopore electrical current signatures,^27^ changes in graphene field effect transistor current,^28^ and shifts in the resonant wavelength of a silicon micro-ring resonator.^29^ Compared to dCas9 assays, nCas9-based assays are less common, but some have utilized nCas9 nicks to initiate strand displacement amplification reactions to detect NAs of interest.^30,31^ Cas9 has also been used to induce isothermal Cas9-mediated exponential amplification reactions (CAS-EXPARs).^30^ In these reactions, a Cas9/single guide RNA (sgRNA) cleaves an ssDNA target, generating a primer that binds to a reaction template containing two primer binding sites flanking a recognition site for a nicking endonuclease. Primer bound to the 3′ end of this template is extended by a polymerase and then nicked at a nicking endonuclease site to create a new site for polymerase-mediated primer extension. This displaces the downstream primer sequence generated by the initial primer elongation event and allows the liberated primer to bind to other reaction templates to repeat this process. CAS-EXPAR can distinguish single nucleotide mismatches between the target and template sequences, which is not possible with other nicking-induced amplification reactions that utilize nCas9, but this capability is restricted to naturally occurring or synthetically generated ssDNA targets (*e.g.*, single-stranded DNA viruses or ssDNA targets generated by reverse transcription of RNA sequences).

#### 2.1.4 Other types of Cas

Cas14 (also called Cas12f)/crRNA complexes recognize ssDNA or ssRNA target sequences and have ssDNA-specific *trans*-cleavage activity. Cas14 is better suited for SNP genotyping analyses than Cas12a since Cas14/crRNA complexes can distinguish single nucleotide mismatches between their target and crRNA sequences with greater precision.^32,33^ Cas14a1 recognition of a target ssDNA sequence also induces *trans*-cleavage of ssDNA without inducing *cis* cleavage of the target ssDNA, allowing continued signal production without a parallel decrease in NA target levels.^33^ This Cas14 feature was used to develop an assay platform (amplification, transcription, Cas14a1-based RNA-activated *trans* ssDNA cleavage, or ATCas-RNA) that detected a bacterial RNA target with 1-attomolar sensitivity and high specificity in milk samples infected with *E. Typhi*, as an example of its performance on complex samples. Another Cas, Cas3, recognizes dsDNA targets and has ssDNA *trans*-cleavage activity, which has been employed to develop the Cas3-Operated NA detectioN (CONAN) assay platform for the detection of SARS-CoV-2 and influenza virus.^34^ Finally, assays based on a class 1 type III CRISPR system that contains multiple Cas protein effectors, including Cas10 that has RNA-activated collateral ssDNA-cleavage activity, and assays based on this system have been developed to detect target miRNAs and SARS-CoV-2 RNA.^35–39^ New types of Cas proteins, which are continuously being characterized, may be a rich source of new tools benefitting future CRISPR-Dx applications.

#### 2.1.5 Kinetics of Cas12 and Cas13 *trans*-cleavage activity

It was initially reported that Cas12 and Cas13 *trans*-cleavage reactions were diffusion limited and had turnover rates of approximately 1000 targets per second,^4,40^ but this value was later corrected to approximately 1 target per second for Cas13b^41^ and 17 targets per second for Cas12a^42^ after the inconsistency of this data was reported by Ramachandran and Santiago.^43^ Extensive studies^44,45^ performed since then have also reported similarly slower catalytic kinetics. These slow reaction rates restrict the achievable limit of detection, as a signal above the reaction background cannot be generated in a reasonable time frame when the target concentration is low.^46^ As a result of the inconsistencies discovered in initial data, it was proposed that CRISPR assay reports should include kinetics data and more experimental detail to allow them to be reproduced and evaluated.^47,48^ This is particularly important for assays that use Cas *trans*-cleavage activity to detect of low concentration targets without a preceding NA amplification (NAA) procedure.

## 3 CRISPR/Cas-based applications and their potential for adaptation to POCTs

The development of CRISPR-Dx assays typically follows a standard workflow. First, using synthetic or mock samples, the researcher tests signal amplification and readout methods to evaluate the assay's sensitivity and specificity for its biomarker target and decides whether a different approach, or a target preamplification step, is necessary to achieve desired performance. Next, isolation procedures employed to isolate target material from clinical specimens (if used) are revised to improve target detection and simplify the assay workflow, as necessary. Finally, successful assays can then be adapted for incorporation into multiplex assays or point-of-care devices. We discuss each of these steps in the following sections.

### 3.1 Preamplification methods

CRISPR-based assays are often employed to detect low abundance targets since Cas/crRNA complexes can exhibit high affinity for their target sequences and their cleavage activity can often be used for signal amplification. However, slow enzyme kinetics and background signal may still require an assay to employ a target NAA step to improve the LOD for scarce NA targets or to decrease the reaction time required to produce a detectable signal for a chosen assay readout method. NAA-based CRISPR assays often report LODs in the attomolar range while LODs reported for non-NAA CRISPR assays typically fall in the femtomolar range. Isothermal NAA methods are frequently selected for this step, since they do not require a thermocycler and often function effectively over a relatively broad temperature range, making them more suitable for use in low-resource settings. RPA and LAMP are the most popular choices, but some assays have also employed RCA,^49,50^ strand-displacement amplification (SDA),^51,52^ exponential amplification reaction (EXPAR),^53,54^ and NA sequence-based amplification (NASBA).^55^

CRISPR-based assays that combine an NAA step and CRISPR reaction in one tube are highly desirable for low-resource settings and can simplify sample handling procedures, reducing the risk for operator error and for cross-contamination. This combined approach, however, can be challenging as it requires simultaneous optimization of the NAA and CRISPR reactions for target sensitivity and specificity, and these reactions can have distinct preferences for reagent composition, buffer system and reaction temperature. For example, LAMP prefers higher temperature (55–70 °C) than tolerated by standard Cas proteins, thus integrating LAMP and CRISPR into one tube assays can requires the use of thermostable Cas protein variants such as AapCas12b^56^ One-pot reactions can employ either sequential^57,58^ or parallel^24,59^ NAA and CRISPR reactions. Sequential one-pot reactions are usually achieved by separating the CRISPR reagents from the NAA reaction until after its completion and then using a physical force, from centrifugation or shaking, to mix these reagents without opening the tube, although interesting alternatives have also been used, namely, a photo-controlled CRISPR–Cas12a reaction^60,61^ and a dynamic aqueous multiphase reaction.^62^ One-pot assays that employ parallel NAA and CRISPR reactions can have simpler workflows but must be designed so that temperature, reagent concentrations, and reagent compositions are optimal for both reactions so that CRISPR activity cleaves NAA amplicons at a rate that does not markedly reduce the sensitivity or kinetics of the assay. One approach to address this is to slow the *cis*-cleavage rate through the use of suboptimal PAMs, although this can reduce assay sensitivity.^63^ A recent study proposed that amplicon cleavage in one-pot assays can be delayed by allowing Cas–crRNA complexes to form during the NAA reaction, instead of adding preformed complex.^64^ This approach exploited the slow association kinetics between Cas12a and its crRNA to reduce competition between the NAA and cleavage reactions by delaying cleavage of the target amplicon and was reported to significantly improve the detection limit. This new assay was also compared with a collection of NAAT–CRISPR–Cas one-pot diagnostics assays that increase sensitivity by mitigating the competition between amplification and detection (for detailed data please go to Table S1†).^64^

Detection of RNA targets in one-pot assays is further complicated by the need to integrate a reverse transcription step. RNA–cDNA heteroduplexes that remain after reverse transcription can also slow NAA initiation to decrease sensitivity, and Feng *et al.* have reported that adding RNase H to eliminate these RNA–cDNA hybrids can improve the sensitivity of RNA detection.^65^

### 3.2 Signal readout

Many different approaches have been used to couple reporter systems to Cas protein activity to enable sensitive detection or quantification of NA targets or other molecules from which NAs can be generated or released as indirect signals. Most of these assays generate optical or electrical signals. Each of these signal transduction methods has strengths and limitations, and the choice of method may depend on whether the assay will be used as a laboratory test or a POCT.

#### 3.2.1 Fluorescence

Fluorescence is the most common readout method for recently reported CRISPR-based assays. A well-known example of this is when an assay that uses Cas12 or Cas13 for target detection employs the collateral cleavage activity induced upon target binding to cut an ssDNA or ssRNA reporter. The cut dissociates fluorophore and quencher molecules at the 5′ and 3′ ends of the reporter, allowing the fluorophore to produce a fluorescent signal when excited by incident light of the appropriate wavelength.^3,20^ But DNA constructs with more complicated secondary structures, such as G-triplexes^66,67^ or G-quadruplexes,^68^ have also been used to generate fluorescent reporter substrates. Studies have also examined the optimal length and sequence composition of such reporters and the relative sequence preference of Cas12 and Cas13 proteins from different sources.^23,44,69^ An advantage of all such reporter-based assays is that the same reporter can be used in CRISPR assays that detect different NA targets, since the collateral cleavage specificity of these enzymes is not affected by the sequence of the NA target, and thus assay specificity can be readily changed by swapping the crRNA used for target detection without the need to alter other assay conditions.

Cas9-based assays that employ Cas9, dCas9, or nCas9 can also employ fluorescence for signal output. SYBR Green I fluorescence, induced upon the binding of this dye to dsDNA, has been used for real-time monitoring of amplified dsDNA products generated in nCas9 assays, including those produced by CAS-EXPAR^30^ and an nCas9-mediated strand displacement amplification approach.^31^ Cas9 assays can also utilize the Cas9-mediated cleavage of ssDNA reporters containing both fluorophore and quencher molecules to produce a fluorescent signal that is proportional to the assay target sequence. But this approach requires that these reporters be hybridized to the complimentary sequence region of a denatured target amplicon, which results in cleavage of both the reporter and its recognition sequence and thus does not allow for signal amplification, requiring that a sequence-specific reporter be designed and synthesized for each assay target.^70^

Bioluminescence and chemiluminescence reporter systems have also been incorporated into CRISPR assays to permit sensitive detection of assay target molecules. For example, one group developed an assay in which the small and large domains of a luciferase reporter protein were fused to dCas9 proteins. The fused products were then used to generate dCas9/crRNA complexes with different target specificities so that their dual binding to a dsDNA target produced a bioluminescent luciferase signal.^25,71^ An RT-RPA SARS-CoV-2 assay using this system was reported to detect SARS-CoV-2 viral RNA at ∼200 copies per μL within ∼20 min.^71^ Chemiluminescence has also been used to enhance the signal output of CRISPR-Dx assays. One group developed a portable CRISPR/Cas13a chip assay (PECL-CRISPR) in which Cas13a recognition of a target miRNA induced the cleavage of an NA construct to generate primer for an EXPAR amplification reaction, and used enhanced chemiluminescent (ECL) signal produced upon intercalation of the “light-switch” dye [Ru(phen)2dppz]^2+^ into the resulting amplicons as the assay readout.^72^ Another group developed a NAA-free Cas12 assay with an ECL readout to detect HPV-16 virus in undiluted human blood samples at an reported LOD of 0.48 picomolar. This group used an approach where target recognition cleaved a ferrocene-tagged thiolated ssDNA quencher to release it from the readout electrode and permit ECL signal production from l-methionine-stabilized gold nanoclusters conjugated to this sensor.^73^ Similarly, another group used a CuS nanoparticle (NP) with a biotinylated ssDNA tether to suppress a Cu^2+^ ion-catalyzed luminol–H_2_O_2_ reaction.^74^ In this assay, the recognition of a target miRNA by a complementary oligonucleotide permitted its ligation to serve as the substrate for an RCA reaction that produced a Cas12 target whose recognition induced the cleavage of the ssDNA tether of the CuS NP. Subsequent addition of AgNO3 then initiated a cation exchange reaction to induce the Cu^2+^ ion-catalyzed luminol–H_2_O_2_ reaction. Bioluminescence and chemiluminescence assays do not require incident light, unlike fluorescence assays that require specific excitation spectra, but instead require other reagents that have the potential to interfere with the reactions that lead to the production of the assay signal.

Real-time and endpoint fluorescent signals can be monitored by instruments, such as plate readers, fluorescent imagers, or spectrophotometers, commonly found in well-equipped research labs or clinical laboratories. Compact devices can also be used to excite the cleaved reporter molecules, producing a fluorescent signal that can then be detected by eye, by a smartphone camera, or by an integrated device. Such devices facilitate POC testing, and handheld readers or integrated systems (*e.g.*, MASTR Pouch^75^) have been developed for this purpose.

#### 3.2.2 Colorimetry

CRISPR-based assays have also employed reactions that produce colorimetric changes, which can be detected by eye or read by portable devices. Many different assay types have been designed to use colorimetry, but lateral flow assays (LFAs) are a particularly common approach since they are easy to use, deliver rapid results, and are inexpensive to produce and distribute.

The SHERLOCKv2 assay^69^ employs an LFA format in which ssRNA reporters with FAM and biotin tags are cleaved by Cas13a/crRNA complexes bound to the target RNA, while intact reporters are captured by streptavidin conjugated to the first line on the LFA strip and detected by gold nanoparticle (AuNP)-conjugated antibodies specific for FAM. Cleavage of the reporter prevents these (AuNP)-conjugated antibodies from binding and allows them to migrate to the second line where they are captured by protein A. Nanoparticle binding produces a colorimetric signal at these two strip regions, and the presence and relative abundance of the assay target is determined by the loss of colorimetric signal at the test line as it shifts to the control line. This LFA AuNP-signal approach has been applied to detect various targets, including SARS-CoV-2,^57,76–78^ HPV,^79^ and cytomegalovirus (CMV) and BK polyomavirus (BKV).^80^ A similar AuNP loss-of-signal readout approach has also been used to detect target-dependent Cas12 cleavage of an ssDNA reporter in proportion to target amplicon abundance.^81,82^ Finally, commercial pregnancy test strips have been employed to detect HPV and SARS-CoV-2, using an approach where target recognition by Cas12a cleaves a dsDNA oligonucleotide sequence that tethers a human chorionic gonadotropin (hCG) protein reporter to an NP that otherwise prevents its transit across, and detection by, the LFA test strip.^11,83^

AuNP loss-of-signal readout approaches are best suited to detect high abundance targets, since, with low abundance targets, it can be difficult to detect minor changes in colorimetric signals by eye or with LFA readout devices. Nonetheless, many different approaches have been employed as CRISPR-assay LFA readouts, including ones that produce positive colorimetric signal proportional to DNA target concentration. For example, one such approach used streptavidin to capture Cas9/crRNA complexes bound to biotinylated assay amplicons at the test line, where they were detected by their hybridization with AuNPs conjugated with horseradish peroxidase and an ssDNA complementary to an exposed crRNA hairpin region, while excess AuNPs were bound to an oligo containing the same recognition sequence that was immobilized at the control line.^84,85^ In both these LFAs, AuNP binding was detected by the colorimetric conversion of the peroxidase substrate 3,3′-diaminobenzidine to produce a colorimetric signal detectable by eye. Similarly, the binding of two dCas9/crRNA complexes to adjacent regions on a target sequence has been used to induce the dimerization of Cas9 fusion proteins containing the N- or the C-terminal domains of HRP to catalyze the oxidation of 3,3-5,5-tetramethylbenzidine (TMB) and produce a colorimetric signal that corresponds to target concentration.^26^

Several CRISPR assays have also used AuNPs to produce and/or regulate their assay signal readouts. For example, one assay used Cas9 cleavage activity and a nicking enzyme to produce target ssDNA for subsequent RCA. Then, it detected the colorimetric signal produced when AuNPs conjugated with ssDNA complementary to the target sequence bound these sequential amplicons to induce their aggregation and enhance their scatter effect.^86^ Another Cas12-based assay used a dsDNA reporter with an ssDNA overhang. This reporter connects AuNPs of different size (20 and 60 nanometer) at each of its termini and has a fluorophore placed immediately before the start of its ssDNA overhang region. Upon target recognition, colorimetric and fluorophore-induced signals can be produced.^87^ In this approach, energy transfer from the smaller of the two AuNPs could stimulate a fluorescent signal from the fluorophore. But this signal was quenched by the larger adjacent AuNP in the intact reporter molecule until its release by Cas12 *trans*-cleavage activity acting on the ssDNA reporter region following target detection, which also shifted the colorimetric signal produced by this reporter.

Cas/crRNA complex binding and cleavage activity can also be coupled to many different colorimetric reactions to produce assay readout signals suitable for target detection or quantification. For example, one group developed a Cas9-mediated SDA assay, where recognition of a concatenated target amplicon sequence by the ssDNA overhang region of a dsDNA oligonucleotide conjugated to a magnetic nanoparticle displaces the dsDNA region to release an ssDNA strand conjugated to glucose oxidase.^51^ The assay signal is then read by removing the magnetic beads and analysing the conversion of a colorimetric substrate by the activity of the glucose oxidase displaced from these beads.

#### 3.2.3 Electrochemical methods

Cas-induced electrochemical current changes are another popular choice for assay signal readout. One early study described the design of an electrochemical CRISPR (E-CRISPR) assay that employed a disposable sensor electrode conjugated with an ssDNA reporter modified with a methylene blue electrochemical tag. In this assay, reporter degradation by Cas12a *trans* cleavage following target recognition decreased the electric current in proportion to target concentration.^88^ A 50 picomolar LOD was reported for targets of human papilloma virus 16 and parvovirus B19, without a preamplification procedure. The same group also evaluated the relative merits of employing Cas9 and Cas12a to induce *cis* cleavage of a methylene blue-modified hairpin ssDNA, complementary to the target ssDNA and conjugated onto the sensor electrode.^89^ In this approach, ssDNA target binding to the complementary hairpin sequence on the sensor increased the distance between the methylene blue tag and the electrode, decreasing the electric current detected by the sensor in proportion to target abundance. This current decrease could be enhanced by incorporating Cas9/crRNA or Cas12/crRNA complexes specific for a dsDNA target generated upon hybridization of the sensor and target ssDNAs, with Cas12a producing a greater effect than Cas9. Similarly, another group designed a sensor electrode that employed streptavidin to capture an ssDNA reporter modified with biotin and FAM at its termini, so that binding of the intact ssDNA reporter to this electrode was detected by the electrochemical activity produced by the binding of a FAM-specific antibody conjugated with glucose oxidase to the reporter.^90,91^ In this assay system, recognition of a target miRNA by its specific Cas13a/crRNA complex activated collateral cleavage of the reporter, which decreased capture of the intact reporter on the sensor electrode surface, reducing antibody binding and its production of the electrochemical signal. Notably, this assay was incorporated into a microfluidic device for rapid detection of target miRNAs in small volume samples. Other groups have also designed microfluidic devices to create assays with electrochemical readouts. For example, one group manufactured a 3D-printed lab-on-a-chip assay to detect SARS-CoV-2 RNA and saliva *via* a multiplexed electrochemical output.^92^ In this approach, saliva was digested on-chip to release both SARS-CoV-2 RNA and protein. SARS-CoV-2 RNA was then captured, reverse-transcribed, and LAMP-amplified, and the resulting amplicons were then employed to induce Cas12a collateral cleavage of a biotinylated reporter. This reporter was then captured by a sensor electrode functionalized with complementary ssDNA. The capture of an intact *versus* a cleaved reporter was assessed by the electrochemical signal produced upon substrate conversion by a streptavidin–HRP conjugate bound to the intact reporter.

#### 3.2.4 Other signal transduction methods

Fluorescent, colorimetric and electrochemical detection approaches are the most commonly employed methods for CRISPR assay signal readout, but several groups have used other highly sensitive approaches to detect a target signal, including a signal from assays that do not employ a target amplification step. For example, surface plasmon resonance (SPR) has been used to detect the signal produced by dCas9 (ref. 93) or Cas12a^94,95^ assays, including one assay platform that used a portable system with a disposable SPR-based fiber tip biosensor for the assay readout.^95^ Notably, it was reported that this system could detect a Monkeypox virus target DNA in spiked blood samples, without amplification, in <1.5 hours with an LOD of ∼60 copies per μL. Surface-enhanced Raman spectroscopy (SERS) has also been used as a readout for NAA-free assays. In one such assay, dCas9/crRNA complexes coupled to gold-coated magnetic nanoparticles were employed to capture target DNA, which was then incubated with the DNA-intercalating Raman reporter methylene blue and detected with a methylene blue-mediated SERS signal.^96^ Although SERS permits highly sensitive detection of low abundance targets without a preamplification step, its instrument costs and other requirements limit its utility as a means for assay readout, particularly in low-resource settings where such diagnostic tests may be in demand. Signal readout approaches using graphene field-effect transistors,^28,97–99^ solid-state nanopores,^27,100–102^ and hydrogels^103–105^ have also been used with some degree of success in CRISPR signal transduction. Several groups have also employed portable personal glucose meters as CRISPR assay readouts. In these assays, Cas activity releases invertase from a solid-phase support by cleaving its nucleic acid tether, and released invertase activity in the assay supernatant can be read by glucose production after the addition of a fixed amount of sucrose.^106–112^

### 3.3 NAA-free methods

Standard LFA and fluorescence-based CRISPR assays that do not employ NA preamplification steps may be limited to picomolar sensitivities due to the slow Cas enzyme kinetics and by background noise derived from nonspecific probe degradation or incomplete quenching systems that employ fluorophore-quencher probes for signal readouts. However, adding an NAA step increases the assay run time and the risk for non-specific amplification or cross-contamination. Combining isothermal amplification and CRISPR into one-pot assays can reduce cross-contamination, but this requires simultaneous optimization of both reactions, which increases their difficulty. Researchers have therefore pursued many strategies to develop NAA-free assays, as discussed in recent reviews.^12,113,114^

One promising approach is to design a feedback circuit where signal production is coupled to Cas activity, as this can transform a linear Cas cleavage response into an exponential signal. One example of this approach is the CRISPR–Cas-only amplification network (CONAN).^115^ In this platform, recognition of the target DNA activates transducer 2, comprised of Cas12a and a dsDNA probe, and the resulting Cas12a *trans*-cleavage activity liberates a caged crRNA that can then target transducer 2, creating a positive feedback circuit that generates an exponentially increasing fluorescent signal. This approach may be promising for POCTs since it employs a single enzyme, requires one 37 °C reaction step, and can reportedly achieve attomolar detection sensitivity. Autocatalysis-driven feedback amplification strategies have also been reported for Cas13-based assays for RNA targets that use hairpin RNA^116^ or a three-stranded RNA probe^117^ to mediate signal amplification.

Another popular approach to overcome the Cas kinetics limitation is to employ picoliter or femtoliter droplets or a microchamber to reduce the reaction volume and thus effectively increase the reactant concentration.^118–122^ Such assays can provide greater sensitivity and quantitative results for targeted molecules, but setup of their spatially confined platforms requires additional equipment and training. To make such assays suitable for low-resource settings, researchers must integrate the reaction setup and signal readout into a user-friendly microfluidic system. Other methods, such as protein engineering for faster kinetics^23^ and using multiple Cas/crRNA complexes to detect different regions of the target,^58,123,124^ have also been explored for NAA-free assays.

As discussed in 2.1.5, there is also controversy regarding LODs attained by some assays that do not use preamplification steps, as some reported LODs were deemed to be physically impossible.^47^ NAA-free assays are frequently reported at the early stages of development. However, future assays, especially those intended for use in low-resource settings, need to report their adherence to the REASSURED criteria and have their performance verified against a reference assay. For example, a a one-step NAA-free Cas12a-aptamer fluorescent detection strategy for prostate-specific antigen (PSA) was compared to enzyme-linked immunosorbent assay (ELISA), surface plasmon resonance, ECL, SERS, electrochemistry, and radioimmunoassay approaches to evaluate their relative performance, procedure times and complexity and sample pre-treatment and equipment requirements.^125^ The study reported that the LODs and working ranges for this assay and ELISA were 0.16 ng ml^−1^ and 0.35–5 ng ml^−1^*versus* 0.19 ng ml^−1^ and 0.31–20 ng ml^−1^, respectively.

### 3.4 Sample processing

Diagnostic assays can analyse a variety of specimen types (*e.g.*, nasopharyngeal swabs, blood, saliva, and urine), which must often be processed prior to their analysis. Traditional NA extraction approaches can be cumbersome and time-consuming, and they can also introduce a contamination risk. Some CRISPR applications do not require purified NA samples, however, and simple NA release protocols, such as the widely adopted heating unextracted diagnostic samples to obliterate nucleases (HUDSON) method^21,126^—which uses heat and chemicals to inactivate sample nucleases and lyse viral particles—have been developed to streamline sample processing for these assays. As an alternative, other studies have used proteinase K to degrade sample proteins and enhance the release of NA targets.^127^ Such streamlined processing methods are not appropriate for all assays and targets, since NA purification and concentration steps are often needed to sensitively detect low-concentration NA targets in complex samples. NA extraction methods that use magnetic beads^56^ or membranes^128^ as affinity matrices for NA capture have been developed as rapid NA isolation and concentration methods suitable for use with, or integration into, CRISPR assays, including assays where the NA extraction and purification steps are integrated into microfluidic devices.^129–131^

### 3.5 Multiplex CRISPR assays

Multiplexed CRISPR-Dx assays could have substantial value for clinical applications, since it is often necessary to simultaneously monitor several biomarkers in a sample to obtain accurate information for diagnosis and treatment decisions or to screen for multiple target pathogens in epidemiologic surveillance studies, but these assays are subject to technical challenges. Multiple Cas/crRNA complexes can be used in one assay to recognize different targets, but sensitive detection methods often employ Cas *trans*-cleavage activity for signal amplification, which can limit the discrimination of signals arising from different markers in a sample. SHERLOCKv2 has attempted to address this issue by employing four Cas proteins (PsmCas13b, LwaCas13a, Cca-Cas13b, and AsCas12a) that have different preferences for NA targets (ssDNA or dsDNA) and *trans*-cleavage sequences (poly AU, UC, AC, or GA),^69,132^ although this approach is limited by the number of Cas proteins with desired target and substrate preferences and thus is not scalable. Massively multiplexed parallel detection can, however, be achieved by using an array of crRNAs with a single Cas protein type in a microfluidic-based labelled nanodroplet assay *via* the combinatorial arrayed reactions for multiplexed evaluation of nucleic acids (CARMEN) platform, which can test more than 4500 crRNA-target pairs on a single array.^133^ This assay causes nanodroplet pairs containing CRISPR detection reagents and amplified targets to fuse in a microwell array, detects the specific fluorescent dyes added to each distinct Cas/crRNA and amplification reaction to identify the nanodroplet pairs present in each well, and reads the fluorescent signal produced by Cas13a *trans*-cleavage of the reporter in each well. This assay was used to detect and distinguish 169 human viruses, including SARS-CoV-2 variants, in eight samples. A subsequent study developed a revised assay, which detected a panel of 21 respiratory viruses including SARS-CoV-2, other coronaviruses, and individual influenza H and N subtypes, and also found that this approach could identify an array of HIV mutations associated with drug resistance.^134^ Other multiplex assays using spatially separated reactions have also been designed to detect HPV,^67^ miRNAs,^70,91^ foodborne pathogens,^135^ SARS-CoV-2,^136^ and other targets.

### 3.6 Microfluidic-based CRISPR assays and wearable devices

Most if not all CRISPR-Dx procedures, including steps of NA extraction, amplification, Cas reaction, signal transduction, and readout, can be integrated into custom microfluidic devices, but this has been recently reviewed elsewhere^7,8,137^ and will not be discussed in detail here. Notably, however, a few CRISPR-Dx assays have been fully integrated into wearable devices.^138,139^ One group developed a face mask with a lyophilized CRISPR sensor, designed to detect SARS-CoV-2 in respiratory droplets, consisting of a push-button controlled fluid reservoir; an aerosol collection membrane; a sample processing area containing spatially separated lyophilized lysis, RT-RPA, and CRISPR reagents; and an LFA signal readout sensor.^139^ In this device, pressing the button on the fluid reservoir caused captured material to flow through the lyophilized reagents to initiate a room-temperature RT-RPA CRISPR reaction that could be read within 90 min and had a SARS-CoV-2 detection limit comparable to that of qPCR. Another group designed a wearable CRISPR microneedle patch that could directly interact with the interstitial fluid of the skin to allow extended (10 day) online detection and monitoring of Epstein–Barr virus (EBV) DNA and changes in cfDNA associated with tissue injury accompanying sepsis and donor kidney rejection.^138^ The patch detected changes in the electric current in the microelectrode upon interaction of a microneedle dCas9/crRNA-activated graphene interface with its NA target. These wearable approaches may have significant potential for rapid, real-time monitoring of patients at risk for specific disease conditions and could possibly assess the effectiveness of therapeutic approaches initiated to treat these conditions.

## 4 Application of CRISPR-based molecular diagnostics for infectious diseases

### 4.1 COVID-19

**Fig. 3:**
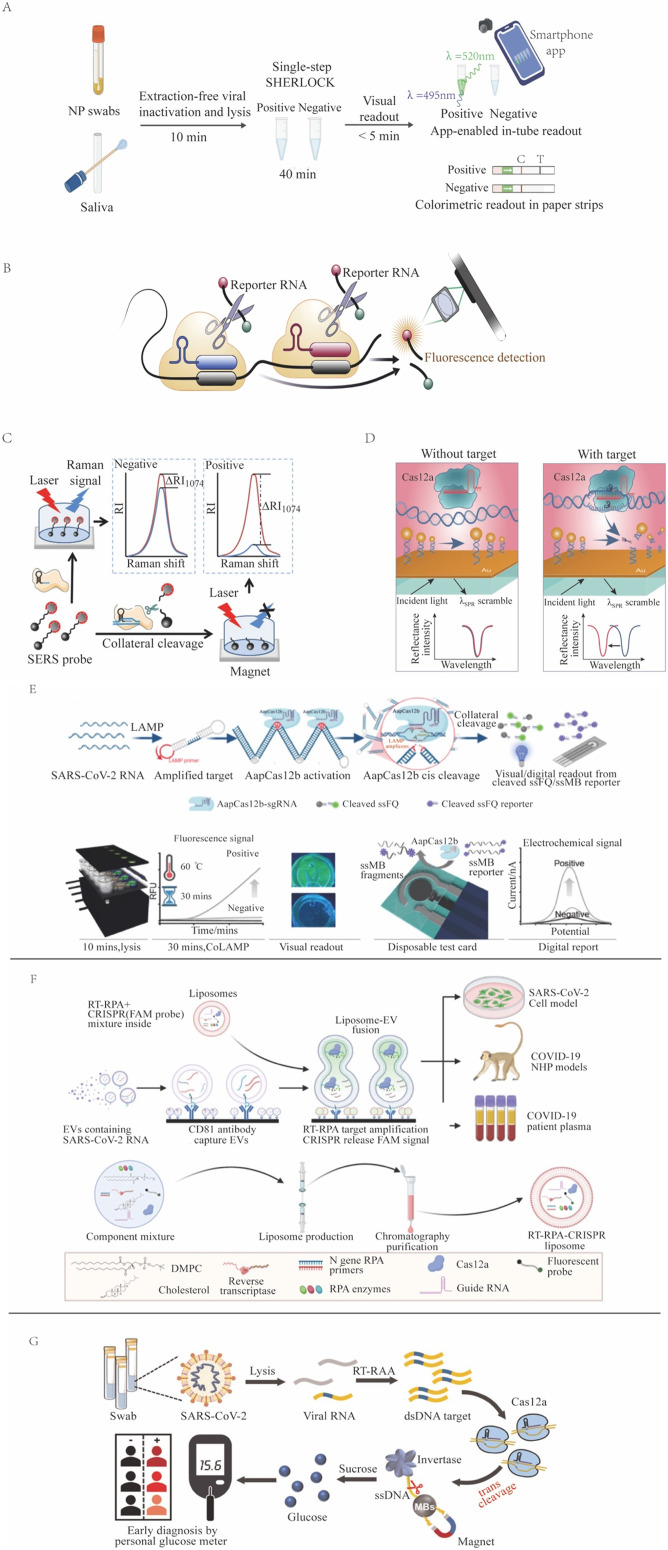
CRISPR-based SARS-CoV-2 detection methods. (A) SHERLOCK employs Cas13a target recognition to cleave a reporter, detecting signal from unextracted diagnostic samples (adapted from ref. 126 with permission). (B) An NNA-free Cas13-based assay employs multiple CAS13/crRNA complexes with different specificities to enhance target signal (adapted from ref. 144 with permission). (C) SERS-CRISPR assays use Cas12a recognition of a DNA target to cleave a quenched reporter and yield SERS signal (adapted from ref. 152 with permission). (D) A Cas12a assay where target recognition is used to cleave a modified ssDNA reporter bound to a sensor to induce a surface plasmon resonance signal (adapted from ref. 94 with permission). (E) CoLAMP assays employ AapCas12a recognition of a LAMP-amplified target to induce *trans* cleavage of quenched reporters, which can produce fluorescent or electrochemical assay readouts (adapted from ref. 153 with permission). (F) A liposome-fusion RT-RPA CRISPR assay that detects SARS-CoV-2 RNA-positive extracellular vesicles in plasma after their affinity capture on an assay well surface (adapted from ref. 154 with permission). (G) A Cas12a assay employs target recognition to cleave a ssDNA tether linking invertase to a magnetic bead. After magnetic bead removal, invertase-mediated conversion of sucrose to glucose can be read as an assay signal with a personal glucose meter (adapted from ref. 106 with permission).

CRISPR-Dx assays have the potential to become POCTs that provide rapid and accurate results for on-site screening and diagnosis.^140^ One successful example is the use of CRISPR in the detection of SARS-CoV-2 viral RNA (Fig. 3) during the COVID-19 pandemic, as has been reviewed elsewhere.^141,142^ One group employed the Cas13-based SHERLOCK system to detect NAA of SARS-CoV-2 viral gene targets (spike, nucleoprotein [N], and replicase polyprotein 1ab) on a lateral-flow readout system with an internal control to detect ribonuclease contamination events that could produce false-negative results.^143^ This LFA had an approximate 1 hour sample-to-result time, a reported LOD of 42 copies per reaction, a diagnostic sensitivity of 97%, and a specificity of 100% upon visual inspection of its colorimetric signal. A subsequent NAA-free fluorescent CRISPR Cas13a assay, read by a mobile phone microscope, reported an LOD of 100 copies per μL after 30 min,^144^ but this result was questioned due to its unlikely kinetics and high senstivity.^47^ Similarly, another group developed a RT-LAMP and CRISPR Cas12a-based DETECTR assay targeting the SARS-CoV-2 nucleoprotein E and N genes, which had a 45 min sample-to-result time and an LOD of 10 copies per μL.^76^ Initial tests used nasal swab samples, but CRISPR/Cas12a-based assays work not only for nasal swab samples^145^ but also for longitudinal plasma samples^146^ and for multiple systemic tissues,^147^ having a versatility that improves diagnosis. A saliva test was also developed; this smartphone-read CRISPR/Cas12a-based test for SARS-CoV-2 subsequently demonstrated a 15 min sample-to-result time and an LOD of 0.38 copies per μL, lower than the LOD of the RT-PCR reference assay.^148^ Subsequent CRISPR assays for SARS-CoV-2 were also developed using crRNAs that targeted signature mutations of the alpha, beta, delta, and omicron SARS-CoV-2 variants.^149–151^

Several one-pot SARS-CoV-2 CRISPR-Dx assays have also been developed as POCTs that have also demonstrated lower limits of detection than the RT-PCR reference assays. For example, the RT-free one-pot enzyme-catalyzed RCA-assisted CRISPR/FnCas12a detector (OPERATOR) assay^155^ integrated ligation-mediated RCA and FnCas12a reactions with a fluorescent LFA readout to achieve a 30 min sample-to-result time with an reported LOD of 0.081 copies per μL. The CRISPR-based one-pot loop-mediated isothermal amplification (CoLAMP) assay reported slightly lower sensitivity (0.5 copies per μL with a 40 min sample-to-result time), but balanced the LAMP and CRISPR reactions to deplete the assay target and reduce the risk for subsequent aerosol contamination.^156^

### 4.2 Tuberculosis

**Fig. 4:**
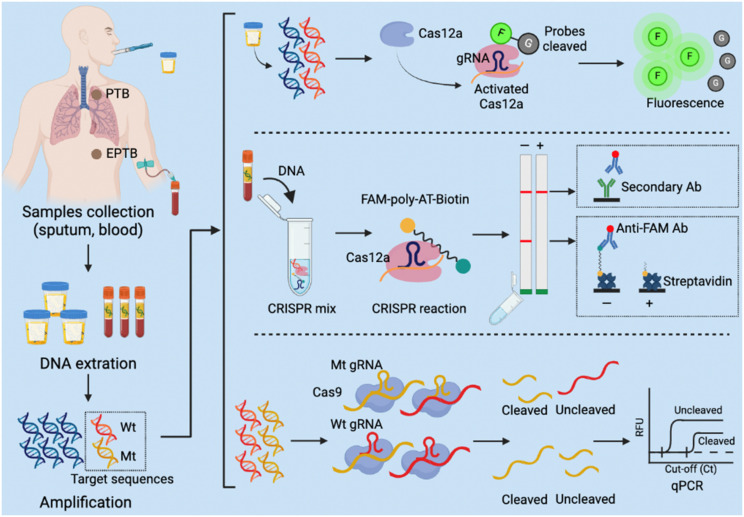
CRISPR-based diagnostic (CRISPR-Dx) assays for *Mycobacterium tuberculosis* detection. PTB: pulmonary tuberculosis; EPTB: extrapulmonary tuberculosis.

Several CRISPR assays have been developed to diagnose tuberculosis (TB), which remains a leading cause of death worldwide but is significantly underdiagnosed by current screening approaches and assays.^157^ Most individuals who develop *Mycobacterium tuberculosis* infections after exposure to TB develop an asymptomatic latent TB infection that can progress to active pulmonary TB or extrapulmonary TB disease at any point in their lifetime,^158^ and current screening methods have limitations, making it difficult to identify and treat new TB cases (Table 1). In 2023, the WHO recommended that TB household contacts, HIV-infected individuals, and other high-risk groups be screened for TB by rapid TB diagnostics (*e.g.*, Xpert MTB/RIF and Xpert Ultra).^159^ But these molecular diagnostic tests are expensive and difficult to implement in low-resource settings. Rapid, sensitive, and user-friendly POCTs are thus urgently needed to improve TB diagnosis and identify drug- resistant TB cases. CRISPR-based molecular diagnostics integrated into POCT platforms have the potential to meet this demand (Fig. 4), and several methods have been developed to date that have sample-to-result times of 2 hours or less and limits of detection that are similar to, or lower than, the Xpert reference assay (Table S1†). The sensitivity estimates of these assays range from 79% to 97.2% and the specificity estimates, from 95.2% to 100%, approaching or exceeding those obtained with Xpert. One clinical study is evaluating a CRISPR-based test using sputum or bronchoalveolar lavage fluid from individuals with suspected pulmonary TB (NCT04074369). CRISPR-based TB diagnostics can also be applied to analyse serum and plasma samples, with at least one study reporting that testing of such samples can effectively diagnose extrapulmonary TB, paucibacillary TB, and paediatric TB cases with and without HIV-1 coinfection, which are challenging cases to diagnose by the current reference methods.^160^ Notably, serum levels of *M. tuberculosis* cfDNA decreased after treatment initiation in this study, suggesting the CRISPR-Dx assays that detect *M. tuberculosis* cfDNA levels in serum could not only increase the coverage of TB screening efforts, but also permit rapid evaluation of treatment efficacy.

**Table 1:** Advantages and limitations of different methods for tuberculosis detection

Diagnostic methods	Advantages	Limitations	Ref
*Mtb* culture and drug susceptibility testing	• Gold standard	• Time consuming: 10–21 days	161
	• High specificity >99%		
AFB staining	• Rapid, practical	• Low sensitivity	162
	• Sputum	• Unable to differentiate different strains	
Tuberculin skin test	• Low cost	• Cannot distinguish latent and active TB	163
	• Widely used, practical	• Low sensitivity in an immune-compromised individual	
		• Influenced by BCG exposure or other atypical mycobacteria	
		• 2–3 days	
IGRA	• Blood	• High cost	164
	• No BCG influence	• 2–3 days	
PCR: line probe assay	• Multidrug resistance	• High cost	165, 166
	• Sputum	• Low sensitivity in paucibacillary patients and non-respiratory samples	
WGS or NGS	• Drug resistance	• High cost	167
		• 3 days	
		• Need well-trained experts	
LAMP: nucleic acid amplification test	• High specificity and sensitivity	• Low sensitivity in paucibacillary patients	168, 169
	• Rapid, practical		
	• POCT		
CRISPR-based detection	• Rapid, practical	• Need further validation and POCT development	160
	• POCT		
	• Blood cfDNA		
	• Available for EPTB and immune-compromised individuals		
	• High sensitivity		
	• Low cost		

Rapid molecular diagnostics are also needed to improve screening efforts to detect and treat the increasing number of drug-resistant and multidrug-resistant TB cases,^157^ and CRISPR diagnostics could play a significant role in meeting this need. For example, a recent study described a Cas9/crRNA-assisted quantitative real-time PCR (CARP) assay designed to directly detect SNPs at two sites in the rifampicin-resistance area that are mutated in 60% to 86% of rifampicin-resistant TB cases.^170^ Another group used a multiplex Cas9-based CRISPR reaction to improve the analysis of 52 candidate genes associated with resistance to first- or second-line anti-TB drugs. This group used crRNAs specific for these genes to cleave the sequences into fragments appropriately sized for an efficient next-generation sequencing analysis to improve the sequencing depth of these target sequences.^171^

### 4.3 Other infectious diseases

To diagnose other infectious diseases, groups have developed CRISPR assays such as LAMP-Cas12a^172^ and recombinase-aided amplification (RAA)-Cas13a assays^173^ that detect HBV DNA in clinical samples and a multiplex RPA-Cas12a/Cas13a assay that simultaneously detects the F3L and B6R genes of monkeypox virus *via* Cas12a and Cas13a cleavage of differently labelled ssDNA and ssRNA reporters.^174^ Multiplexed and portable CRISPR assays have also been developed for SARS-CoV-2, dengue virus, Zika virus, and influenza A and have streamlined workflows that render them suitable for POCTs, and laboratory-based CARMEN microwell assays can perform 1000 tests per chip and detect 169 human viruses.^133^

### 4.4 CRISPR-Dx assays for low-resource settings

POCT CRISPR-Dx assays intended for use in remote or low-resource settings may have requirements not shared by similar tests designed for use in well-equipped clinical laboratories. Assays intended for low-resource settings are normally optimized to reduce material expenses; storage, equipment, and operator requirements; and may also employ decentralized reporting strategies (*i.e.*, connect with a portable smart diagnostic device or smartphone) to facilitate telehealth and epidemiologic efforts. Adapting clinical laboratory CRISPR-Dx assays for use as POCTs in low-resource settings usually involves trade-offs between assay performance and cost, portability, or other considerations that can affect their overall utility. For example, POCTs intended for use in low-resource settings usually employ lyophilized reagents to eliminate or minimize cold-chain requirements during transport and storage, but this comes at the cost of some loss of reagent activity and assay sensitivity. Thus, such trade-offs may produce POCTs that exhibit lower sensitivity and specificity than laboratory-based tests but still retain significant diagnostic value, particularly in the absence of a practical alternative tests. For example, the Cas13-based streamlined highlighting of infections to navigate epidemics, version 2 (SHINEv.2) SARS-CoV-2 assay was designed for use outside centralized laboratories, as it had minimal sample processing and no instrument requirements but a reported LOD of 200 copies per μL.^175^ This assay was also reported to achieve a 90.5% sensitivity 100% specificity when used on unextracted nasopharyngeal swab samples, comparable to rapid antigen tests used to diagnose SARS-CoV-2 infection.

Cost can influence choices for all steps in a POCT workflow, from sample preparation to signal readout. These choices include the selection of assay reagents and the materials and fabrication processes for disposable assay cartridges and their portable readers. One study estimated that per sample costs of several CRISPR- and PCR-based diagnostics intended for use in low-resource settings range from US$7 to $20, although it was not clear if the estimate included laboratory instrumentation and infrastructure costs.^176^ Several groups have attempted to develop inexpensive CRISPR-Dx POCTs. For example, one study described LAMP/CRISPR HPV assay that employed a gold leaf electrode to obtain a sensitivity of 10^4^ copies per test and cost ∼$2.30 per test.^177,178^

The minimally instrumented SHERLOCK (miSHERLOCK)^128^ POC saliva assay for SARS-CoV-2 and its mutations was reported to have a LOD of 1240 copies per mL and cost $15 per test, although this could be reduced to $11 per test by reusing its electronics and heaters. The major assay expense (∼$9) derived from its use of commercial enzymes (RPA and reverse transcriptase) which should be reduced at scale. Another SARS-CoV-2 assay, the Cas9-based FnCas9 editor linked uniform detection assay (FELUDA), had a reported $7 per test cost,^179^ but used PCR for target amplification and thus required a thermocycler.

Incorporating CRISPR assays into microfluidic devices made using inexpensive materials and manufacturing methods can substantially reduce assay costs. For example, one study has reported that a compact, fully-integrated thermoplastic cartridge can function as an automated device to perform a CRISPR-based SARS-CoV-2 assay,^129^ and most assay costs derived from the reagents ($7.84) rather than this cartridge ($0.30). However, this assay was still read by a relatively large and expensive portable device, which could limit its utility in low-resource settings. Several groups have attempted to address similar instrumentation issues by using inexpensive portable devices, including smartphone cameras or smartphone-coupled devices, as assay readers. For example, one group developed a finger-actuated microfluidic biosensor with a one-pot multiplex NAA/CRISPR assay for seven common foodborne pathogens where the assay's fluorescent signal was captured and analysed with a smartphone device and app.^135^ This assay had a reported LOD of <500 CFU mL^−1^ with a 1 hour sample-to-result time, a microfluidic chip per-test cost of <$4, and an assay device cost of $150, excluding the smartphone itself. Microfluidic/electrochemical paper-based analytical devices are also of interest because paper is inexpensive, portable, and disposable,^180,181^ although high-quality paper may be costly in low-resource settings.

Table 2 summarizes efforts to develop assay workflows suitable for use in low-resource settings.^175^ However, there are currently no commercial CRISPR-Dx POCTs approved for use in low-resource settings. SHERLOCK- and DETECTR-based assays for SARS-CoV-2 were authorized by the FDA for emergency use, as was the Cas9-based FELUDA^179^ in India, but these assays are for certified laboratories only.

**Table 2:** Efforts to develop assay processes suitable for use in low resource settings

Objective	Effort	Representative studies
Simplified sample processing	Heat-mediated nuclease inactivation and NA release	Myhrvold^21^ Arizti-Sanz^126^
	Magnetic bead-assisted NA purification	Joung^56^
	PES membrane-mediated NA concentration	de Puig^128^
	Integrated microfluidic sample processing	Chen,^129^ Wu 2021 (ref. 130)
Simplified assay workflow	One-pot amplification and detection	Ali^57^ Li^24^ Ding^59^ Chen^60^ Feng^65^
	NAA-free assay approach	Shi^115^ Yang^23^
Simplified signal readout	Lateral flow assays	Gootenberg^69^ Mukama^81^ Tang^11^
	Colorimetric assays	Qiu^26^ Gong^51^ Hu^84^ Wang^85^
	Fluorescence assays with minimal instrumentation	de Puig^128^ Ning^148^ Wang^182^
	Electrochemical assays	Zeng^183^ Han^184^
Improved temperature stability	Lyophilized reagents	de Puig^128^ Nguyen^139^ Arizti-Sanz^175^
Affordability	Reduced instrument cost	de Puig^128^ Wang^182^
	Reduced consumable expenses	Zamani^185^ Chen^129^

## 5 CRISPR-based diagnostics for early detection of cancer

Early cancer detection leads to early intervention and improves survival rates, as seen with successful screening strategies for cervical, breast, and colorectal cancers. However, there were still 1 958 310 new cancer cases and 609 820 cancer-related deaths in the United States in 2023,^186^ and there are expected to be 28.4 million new cancer cases worldwide in 2040.^187^ Further, about 50% of cancers are diagnosed at a late stage where the odds of long-term survival are substantially worse.^188^ It is therefore crucial to implement cancer prevention and early detection strategies, including those that can effectively serve low-resource settings.

CRISPR-Dx POCTs have shown promise for infectious disease diagnosis and have great potential for cancer, as they could help identify high-risk populations to facilitate cancer prevention and screening efforts in low-resource settings. For example, at-home HPV self-sampling could be a future option for cervical cancer screening, but this approach still requires that samples be sent to a lab. This shortcoming could be addressed by the development of at-home tests for early cancer detection, similar to those developed during the COVID-19 pandemic.

Rapid turnaround time is not as essential for cancer diagnosis as it is for infectious disease diagnosis but developing accurate and low-cost assays with minimal training and equipment requirements will be necessary to permit early cancer screening efforts in large populations in low-resource settings. We discuss some potential CRISPR application scenarios below.

### 5.1 Gene mutations

Early-stage cancer diagnosis remains a major challenge for cancer prevention, partly because cancer screening diagnostics have low specificity and sensitivity for protein biomarkers. NA biomarkers (*e.g.*, circulating tumor DNAs [ctDNAs] and miRNAs) have demonstrated potential as biomarkers for targeted therapy, immune-checkpoint inhibitor treatment, and early cancer diagnosis, but their low concentrations can lead to high false-negative rates when analysed by current detection methods. CRISPR-based assays (Table 3) can enhance sensitive detection of NA targets and potentially distinguish alternate SNPs, and at least one group has employed restriction enzymes to provide target specificity. This excision-amplification-synchronous Cas12a-targeted checkout (EasyCatch) assay approach integrates a restriction enzyme to cleave the wild-type site during RPA-mediated amplification of the NA target sequence, allowing preferential amplification of rare mutant alleles against a high wild-type allele background (0.001% mutant) to permit rapid (<1 h) and sensitive detection of these mutations in cancer samples.^189^ Notably, this approach was used to detect two EGFR mutations (e19del and L858R) and several FLT3 mutations (D835Y/H/V/F) associated with resistance to tyrosine kinase inhibitors, indicating its potential to inform cancer treatment decisions. Cas9- and Cas12a-based biosensing can successfully detect EGFR T790M (93.9% sensitivity and 100% specificity) and L858R mutations in plasma and tissues from patients with lung cancer.^155,190^ Given the ability of CRISPR-based assays to detect SNPs and other mutations associated with specific cancers, cancer subtypes, and drug resistance phenotypes, there is significant potential for the development of CRISPR-based POCTs for common cancer-associated NA biomarkers.

**Table 3:** Proposed CRISPR-based approaches for cancer diagnosis

Cas effector	Target	NAA and/or enrichment	Specimens	Cancer	LOD	Sensitivity/specificity	Ref
Cas9	PCA3 and KLK3	RT-RAA	Urine and peripheral blood	Prostate cancer	500 and 50 fg μL^−1^ LNCaP cell RNA	—	191
Cas9	EGFR mutants	PCR	Blood	Lung cancer	10^−3^–10^−4^ DNA dilution	—	192
Cas9	EGFR T790M mutant	PCR	Plasma	NSCLC	<10 copies per mL plasma	93.9%/100%	190
Cas9	EGFR L858R mutant	ICP	Synthetic samples	—	10^−3^ target DNA dilution	—	193
LbCas12a	EGFR L858R mutant	Isothermal amplification	Lung cancer tissues	NSCLC	0.3 copies per μL (0.498 aM) in mock multiplex cfDNA samples	—	122
LbCas12a	TP53 R273 mutations	PCR	Cell lines HNSCC biopsies	HNSCC	3–6% target DNA dilution	—	194
Cas12a	FLT3 D835 mutations	RE-RPA	Blood	AML	10^−5^ target DNA dilution	—	189
Cas13a	EGFR L858R	RPA	Mock cfDNA samples	—	10^−3^ target DNA dilution	—	3
	BRAF V600E						
Cas13, Cas12a, and Csm6	EGFR L858R		Synthetic and liquid biopsy cfDNA samples	NSCLC	—	—	69
	EGFR exon 19 deletion						
	EGFR T790M						
	APC:c.1262G>A						

### 5.2 Oncogenic viral infections

Persistent HPV infections can increase the risk for several cancers, including cervical cancer and head and neck cancers.^195,196^ According to The Global Cancer Observatory (GLOBOCAN) 2020, the incidence and mortality of cervical cancer remain high in multiple countries.^197,198^ And the WHO now recommends using assays that detect HPV DNA as a primary test in cervical cancer screening and treatment approaches.^195^ Streamlined and inexpensive HPV POCTs are thus required to achieve the WHO's screening and treatment targets and to help eliminate cervical cancer as a public health problem.

Several NAA-based and NAA-free CRISPR-based assays have been reported as potential means to detect HPV subtypes in clinical specimens (Table 4). LODs reported for the NAA-based (RPA, PCR, and RAA) CRISPR assays tended to cluster near 1 attomolar, although other reported LODs corresponding to 1 copy/reaction values for their assay volumes (0.03 to 0.26 attomolar) or substantially higher values (240–664 attomolar). Studies that provided results for non-NAA controls reported LODs in the low picomolar range (3–50 picomolar), with a few in the nanomolar range (0.5–10 nanomolar).

**Table 4:** Applications of CRISPR-based assays to detect human papillomavirus

Effector Cas protein	HPV subtypes	LOD	Reported assay time	NAA method	Readout	Sensitivity specificity	Clinical sample	Ref
Cas9	HPV16,18	—	3–4 h	PCR	Fluorescence	—	Cervical mucus exfoliated cells	196
Cas9	HPV16,18	—	3 h	PCR	Fluorescence	—	Cervical mucus exfoliated cells	199
Cas9	HPV16, 18, 33, 35, 45, 51, 52, 56, 58, 59	—	2 h	PCR	Fluorescence	—	Cervical mucus exfoliated cells	200
Cas9	HPV16, 18, 33, 35, 45, 51, 52, 56, 58, 59	—	2–3 h	PCR	Fluorescence	—	Cervical mucus exfoliated cells	201
Cas9	HPV16, 18, 6, 11, 33, 35, 40,45, 51	—	90 min	HRCA	Visual, fluorescence	—	Cervical mucus exfoliated cells	202
dCas9	HPV16,18, 31, 33, 35, 39, 45, 51, 52, 56, 58, 59, 66,68, 73	—	30 min	HCR	Fluorescence	—	Cervical sample	203
LbCas12a	HPV16, 18	No-RPA: 10 pm	1 h	RPA	Fluorescence	—	Anal swab	4
		RPA: 1 aM						
LbCas12a	HPV16	280 aM	3 h	RPA	Fluorescence (using LFA)	—	Plasma	79
	HPV18	240 aM						
LbaCas12a	HPV16	10 copies (0.83 aM)	1 h	RPA	Fluorescence	—	Plasma, cervical swab	62
	HPV18	100 copies (8.3 aM)						
Cas12a	HPV16, 18, 31, 33, 35, 39, 45, 51, 52, 56, 58, 59, 68	100 copies (13 aM) to 500 copies (66 aM)	35 min	RPA	Fluorescence	—	Cytological scrape	204
LbCas12a	HPV16	No sensor: 100 pM	—	RPA	Fluorescence, electrochemical	—	Vaginal swab	205
		Sensor: 1 pM						
LbCas12a	HPV16,18	No-PCR: 50 pM	—	PCR	Fluorescence, LFA	94.7%/100%	Anal swab	66
		PCR: 1 copy per rxn (0.1 aM)						
Cas12a	HPV16,18 and HIV	10^4^ copies (664 aM)	—	LAMP	Electrochemical	100%/89%	Cervical swab	177
Cas12a	HPV16	No RPA: 3 pM	—	RPA	Chemiluminescent, visual image	88.9%/100%	Clinical samples	206
		RPA: 1 copy per rxn (0.03 aM)						
LbCas12a	HPV6, 11, 16, 18, 31, 33, 45, 52, 58	0.26 aM	40 min	RPA	Fluorescence	97.8%/	Cervical swab	67
						98.1%		
LbCas12a	HPV16	No RPA: 17 pM	—	RPA	Chemiluminescent, visual image	87.5%/100%	Clinical samples	207
		RPA: 1 copy per rxn (0.09 aM)						
LbCas12a	HPV16	No RPA: 50 pM	—	RPA	Colorimetric signal on the PTS	—	Vaginal or urethral discharge	11
		RPA: 2 copy per uL (3.3 aM)						
	SARS-CoV-2							
LbCas12a	HPV16,18	No RPA: 20 pM	30 min	RPA	Fluorescence	92.3%/100%	Cervical cell specimen	208
		RPA: 1 aM						
LbCas12a	HPV16,18	No RPA: 0.5 nM	30 min*	RPA	Fluorescence, (microchip+LFA)	—	Cervical swab sample	209
		RPA: 1 aM						
LbaCas12a	HPV16,18	No-RPA: 10 nM	—	RPA	Fluorescence	—	Cervical brush specimen	210
		RPA: 2 aM						
Cas12a	HPV16,18	1 aM	80 min	RPA	Fluorescence	100%/93.8%	Clinical samples	211
LtCas12a	HPV16,18	30 copies (2 aM)	—	RAA	Fluorescence, LFA	90% /96.2% (16)	Sanitary napkin blood samples	212
						92.3% (18)		
Cas12a	HPV16,18	1 copy per μL (2 aM)	—	RAA	Fluorescence	97.1%/100%	Cervical epithelial tissue	213
Cas13a								
LbCas12a	HPV16	50 pM	—	No NAA	Electrochemical	—	No	88
	PB19							
LbaCas12a	HPV16	0.1 pM	—	No NAA	Electrochemical	—	No	214
LbaCas12a	HPV16	3.2 pM	50 min	No NAA	Electrochemical	—	HPV-spiked serum	215
Cas12a	HPV16	1.6 pM	—	No NAA	Photocurrent	—	No	216
Cas12a	HPV16	1.2 pM	—	No NAA	Photocurrent	—	Cervical brush samples	183
Cas12a	HPV16	1 pM	—	No NAA	Photocurrent	—	HPV-spiked serum	217
Cas12a	HPV16,18,52	0.22 pM	—	No NAA	ICP-MS signal	—	Cervical swab sample	218
Cas12a	HPV16	42.9 pM	30 min	No NAA	Lateral flow biosensor	—	No	219
	HPV18							
		0.21 pM						
Cas12a	HPV16	3.2 fM	—	No NAA	ECL	—	No	220
Cas12a	HPV16	8.9 fM	100 min	No NAA	ECL	—	HPV-spiked serum	221
dCas9	HPV 16	—	—	No NAA	Raman spectroscopy	—	No	222
Cas12a	HPV16,18 and HBV	1 aM	20 min	No NAA	Raman spectroscopy	—	No	223
LbaCas12a	HPV18	DNA 10 aM	30 min	No NAA	Fluorescence (digital droplet)	100%/100%	Cervical epithelial cells	224
LbuCas13a	SARS-CoV-2	RNA 100 aM						

Most NAA-free CRISPR assays reported LODs in the low picomolar range (0.1–50 picomolar), consistent with the non-NAA controls of the NAA assays. Lower LODs were reported for assays employing electrochemoluminescent (ECL, 3.2–8.9 femtomolar), digital droplet (10–100 attomolar) and Raman spectrometry (1 attomolar) readouts, but these results appear feasible given the high sensitivity of each of these approaches.

Multiple NAA approaches have been used for the HPV assays. For example, one group developed a CRISPR-associated hyperbranched RCA technique (CART) assay that used two Cas9/crRNA complexes, one specific for the L1 region of HPV16 and the other, for the L1 region of HPV18. These complexes excised the intervening DNA region, which was then ligated to generate a circular DNA target for an isothermal RCA reaction; the amplified target was then detected by agarose gel electrophoresis or a fluorescent signal produced upon dye intercalation.^205^ Another group subsequently developed a CRISPR-typing PCR (ctPCR) HPV assay that employed two Cas9/crRNA complexes recognizing conserved regions in the HPV genome. These complexes excised an NA target region, which was then ligated to linkers containing universal primer sequences to allow multiplex amplification of the corresponding region of multiple HPV strains for subsequent analysis.^201^ Several variants of this assay approach have been developed (ctPCR 2.0, 3.0 and 4.0).^196,200,202^ But neither the CART nor the ctPCR assays were further adapted to formats suitable for POCTs. Cas9 may also not be the best candidate for a CRISPR-based HPV diagnostic due to its potential for off-target cleavage events.^225^ A Cas12a-based DETECTR assay, however, could specifically and rapidly detect HPV DNA with attomolar sensitivity.^4^ CRISPR/Cas12a *trans*-cleavage activity can also be used to degrade synthetic assay probes that produce a variety of signals (fluorescent,^62^ electrochemical,^205^ and colorimetrõic^79^) detectable on LFA or microfluidic chips through visual inspection or by a handheld device. For example, one group employed a Cas12a-based assay to detect HPV DNA targets in diluted plasma samples (without DNA isolation) using a visual LFA readout, which detected positive HPV16 and HPV18 signals in 93% and 30% of the plasma samples from a small cohort of patients with cervical cancer.^79^

Several studies have also used NAA-free CRISPR assays to detect HPV (Table 4), but while these assays can be faster, less expensive, and more streamlined than amplification-based assays, they also often require strategies to increase signal production or detection from low-concentration targets. For example, one group developed a portable photoelectrochemical (PEC) assay for HPV16.^183^ In this assay, target-induced Cas12a *trans* cleavage depleted a catalytic G-quadruplex, alleviating its inhibition of a photoactivated screen-printed electrode, which induced a photocurrent signal that was detectable by a smartphone device. Similarly, another group developed an electrochemical biosensor, in which target-mediated Cas12a *trans*-cleavage activity was employed to cleave a methylene blue-labelled ssDNA reporter from the assay biosensor to induce an electrochemical signal that had a 50 picomolar LOD.^88^ A third group developed a polydisperse droplet digital CRISPR/Cas (pddCas)-based HPV assay that used a standard vortex mixer to generate picoliter droplets, and reported LODs of 10 to 100 attomolar for DNA and RNA targets.^224^ Droplet digital CRISPR/Cas (pddCas)-based HPV assay that used a standard vortex mixer to generate picoliter droplets, and this assay had an LOD of 10 to 100 attomolar.^224^

Clinical laboratory-based PCR tests for EBV are used for diagnosis of nasopharyngeal carcinoma and other EBV-related diseases.^226^ EBV-based screening for nasopharyngeal carcinoma is recommended in high-risk regions.^226^ EBV-based screening for nasopharyngeal carcinoma is recommended in high-risk regions.^156^ One prospective study in Hong Kong^227^ analysed plasma samples from over 20 000 participants by real-time PCR and participants with EBV-positive were retested after 4 weeks. This study determined that 34 of the 309 participants with persistent EBV-positive results later received nasopharyngeal carcinoma diagnoses, while only one individual with EBV-negative results developed nasopharyngeal carcinoma within a year.

Several groups have employed various approaches to reduce the equipment demands for such screening efforts. One group recently developed a Cas12a-based colorimetric assay that permits visual detection of EBV-positive serum samples analysed under nonlaboratory conditions. This assay employed Cas12a *trans*-cleavage activity to degrade an ssDNA region of an oligonucleotide used to cross-link AuNPs that were spiked into RPA reactions of diagnostic serum samples. This degradation reduced the centrifugal precipitation of disrupted AuNP aggregates and produced a colorimetric signal proportional to the amount of AuNPs retained in suspension.^228^ A second group developed an NAA-free microfluidic Cas12a-based digital droplet assay to detect EBV in serum samples.^121^ A third group described a proof-of-concept real-time, wearable Cas9 assay patch, designed to allow NAA-free detection of EBV cfDNA in interstitial fluid.^138^ There is also an ongoing clinical trial to evaluate the performance of a Cas12a-based assay for EBV DNA detection in nasopharyngeal brushing and plasma samples (NCT05447169).

### 5.3 miRNAs

Small (19 to 25 nucleotide) noncoding miRNAs have also revealed promise as blood-based biomarkers in cancer screening applications.^229^ For example, results from a large, randomized trial have indicated that low-dose computed tomography and serum miRNA results can predict lung cancer risk.^230^ Several miRNAs (miR-155, miR-197, and miR-182) have potential for early detection of lung cancer, and ultrasensitive electrochemical biosensors have been established, as reviewed by Shaterabadi *et al.*^231^ Several groups have also established biosensors for CRISPR-based assays to detect miRNA and to increase specificity and provide platforms for POCT applications, as summarized in a recent review.^232^ For example, one group used an array of femtoliter chambers for NAA-free detection of an RNA target by Cas13a cleavage of a quenched fluorescent reporter in a digital droplet assay, achieving femtomolar limits of detection for multiple SARS-CoV-2 RNA targets.^233^ This assay should be adaptable for the detection of miRNA biomarker targets; however, it was read by a plate reader and may not work for low-resource settings or a POCT format. This assay approach also remains substantially less sensitive than CRISPR assays employing amplification steps, such as an amplification-based CRISPR assay using a Cas12a-based chemiluminescence biosensor, which detected miRNA-21 at an LOD of 16 attomolar (ref. 74) and may be more readily adapted to a POCT. Differential miRNA expression signatures may have greater diagnostic value than the evaluation of individual miRNAs, and biosensors that permits multiplex detection of several distinct miRNAs have been developed. For example, one group developed an NAA-free CRISPR/Cas13a-based microfluidic assay for eight miRNAs, including two miRNAs dysregulated in paediatric medulloblastoma.^91^ None of assays described above are ready for use as clinical applications or POCTs, as all of them require additional refinement to stabilize their reagents, followed by analytical and clinical validation studies, before they are ready for adoption as diagnostic tests.

## 6 Limitations and perspectives for future development

New CRISPR-based POCTs for infectious disease and cancer screening, diagnosis, or disease management should be user-friendly, employ minimally invasive or non-invasive diagnostic specimens, and provide rapid and clinically valid results to guide healthcare decisions. CRISPR assays using well-established POCT methods to provide user-friendly readouts (*e.g.*, LFA or personal glucose monitor results) may be best suited for self-monitoring applications (perhaps for individuals with a suspected disease condition) or for clinical decisions by healthcare personnel in low-resource settings. But no single approach is universally appropriate, as different assay designs may have distinct advantages and disadvantages for different specimens or types of NA biomarkers. Assay suitability can be influenced by sample processing and analysis workflows, readout formats, sensitivity and specificity, data interpretation and reporting demands, and sample-to-result times. Therefore, new CRISPR-based POCTs for specific diseases and infections must take these all considerations into account during the initial assay design stage.

Many CRISPR assays use a previous or parallel target amplification reaction to enhance or permit the detection of low-concentration NA targets in clinical specimens. CRISPR assays that amplify and analyse targets in parallel are generally more suitable for use in POCTs than those that require a target preamplification procedure, unless the amplification and analysis reactions can be integrated into a single device to avoid sample manipulation, which can lead to assay workflow errors and inaccurate results. Integrating these two reactions into the same device or well can require careful optimization of the sample processing workflow, assay reagents, and reaction conditions to maximize sensitivity and accuracy of the final POCT. However, even with such careful optimization, assays that employ single reactions for target amplification and CRISPR detection usually have decreased sensitivity, and thus there is frequently a trade-off between sensitivity and simplicity when designing CRISPR assays for POCTs.

Although CRISPR assays without a target amplification step often lack satisfactory sensitivity, these assays can sometimes have acceptable sensitivity when used to detect more abundant NA targets or when coupled with ultrasensitive readout approaches, such as SERS. But even when sensitive readout approaches are applied to detect low concentration targets, their expense and support requirements usually preclude their use in low-resource settings or in POCTs. It may be possible to improve the detection sensitivity of NAA-free assays by identifying, selecting, or engineering new Cas proteins with enhanced binding affinity and *cis*- or *trans*-cleavage activities, but this would require significant effort, and the activity and sensitivity increases that can be achieved through these approaches are not clear.

CRISPR-assays that accurately quantify an NA target may offer significant clinical value by providing a means to estimate the target's abundance in a sample from a patient or an infectious agent, but accurate measurements typically require the use of standard curves or laboratory equipment, which are not feasible for low-resource settings or for POCTs. For example, several groups have developed CRISPR-based droplet assays to quantify NA targets,^119,121,224^ but analysis of these assays requires the use of specialized instruments and costly consumables.

New software for improving the selection of conserved genome sequence regions, amplification primers, and crRNA sequences suitable for use in NA-based diagnostics could streamline assay development. Recent research indicates that a machine learning-based system can automate these processes to significantly improve assay design; one such method required only 2 hours to design CRISPR/Cas13a-based diagnostics for 1933 virus species that infect vertebrate hosts.^234^ Specifically, this method searched the target virus genomes, identifying and scoring potential amplicon regions by their predicted amplification potential and the relative activity of optimal amplification primer sets designed to provide high coverage of sequence diversity occurring at their recognition sites. This and similar approaches could greatly improve the selection of NA targets.

Robust analytical validation studies that include synthetic samples (*e.g.*, target material spiked into a diagnostic specimen obtained from a heathy individual) or real clinical samples are critical to identify assays that have the best potential for success prior to performing clinical validation studies. Such analytical validation studies, however, are often complicated by the lack of accepted internal standards for POCTs or *in vitro* diagnostics. Subsequent clinical validation studies should also analyse samples obtained from patients and controls that reflect the diagnostic complications encountered in the target population, have sufficient power to provide reliable estimates of diagnostic sensitivity and specificity, and include a direct comparison to a reference assay. Most studies describing the development of new CRISPR-based diagnostic assays analyse samples from small case–control studies or from synthetic samples, neither of which may accurately reflect the confounding factors present in target patient populations, and thus have a reduced chance to successfully translate into a clinical application.

Single-visit screening and diagnosis approaches that use noninvasive sample types are preferred to counteract the trend toward more complex diagnostic tests requiring high-throughput sequencing approaches, which can limit testing capacity and coverage of affected patient populations.^235^ New developments allowing for highly sensitive and specific detection of NA biomarkers in minimally invasive or noninvasive liquid biopsy specimens using one-step, quantitative, and multiplexed CRISPR-Dx POCTs^236^ have the potential to meet this demand for simpler and more broadly available diagnostics.

## Data availability

No primary research results, software or code have been included and no new data were generated or analysed as part of this review.

## Author contributions

X. Q. and T. Y. H. conceived and designed this review. X. Q. and Q. X. drafted the manuscript. X. Q., C. J. L. and T. Y. H. revised the manuscript. All authors approved the final manuscript.

## Conflicts of interest

There are no conflicts to declare.

## Supplementary Material

LC-024-D4LC00340C-s001
