# Supplementary material for: CRISPR for companion diagnostics in low-resource settings
Source: Lab Chip. 2024 Sep 5;24(20):4717–40. doi: 10.1039/d4lc00340c (PMC11393808; doi:10.1039/d4lc00340c)
Supplement: LC-024-D4LC00340C-s001 [file LC-024-D4LC00340C-s001.pdf]

Supplementary Information (SI) for Lab on a Chip.
This journal is © The Royal Society of Chemistry 2024

Table S1 CRISPR-based diagnostics for TB and drug-resistant TB

Effector	Strategy	Target	DNA extraction	Sample	Time	LOD	Sensitivity	Specificity	Ref
Cas12a	A recombinase polymerase amplification (RPA) step with CRISPR-based detection	*Mtb* IS6110	A combination strategy of beads beating, chemical lysis, and heating	Sputum	1.5h	5 copies/µL	79%	98%	[237]
Cas12b	TB-QUICK: loop-mediated isothermal amplification (LAMP) and CRISPR/Cas12b detection	*Mtb* IS6110	QIAamp Blood Mini Kit and QIAamp Circulating Nucleic Acid Kit	Pulmonary (sputum, bronchoalveolar lavage fluid) or plasma samples	2h	1.3 copy/µL	Pulmonary: 86.8% Plasma: 41.2% for AFB-positive and 31.7% for AFB-negative patients	95.2%	[238]
Lba Cas12a	CRISPR detection of circulating cell-free Mycobacterium tuberculosis DNA in adults and children, including children with HIV	*Mtb* IS6110, esxB, gyrB	The Quick-cfDNA Serum & Plasma	Serum	2h	0.06 copy/µL	68%-100%	-	[160]
LwCas13a	A sensitive Mycobacterium tuberculosis (MTB) complex polymerase chain reaction (PCR)-CRISPR/Cas13a detection method (CRISPR-MTB)	*Mtb* IS1081	Conventional DNA extraction	Sputum, BALF, and pus samples		1 target sequence copy/µL	97.2%-100%	95.5%	[239]
Cas9	A CRISPR/Cas-9-mediated fluorescent strategy utilizing fluorescence resonance energy transfer (FRET)	High-variable region of M. tuberculosis 16S rDNA fragment	-	Simulated sputum samples	2h	20 CFU mL^{-1}	-	-	[240]
Cas12b	CRISPR/CRISPR-associated 12b nuclease CRISPR/Cas12b-based multiple cross displacement amplification technique (CRISPR-MCDA)	*Mtb* IS6110	Genomic DNA extraction kits	Sputum	70min	5 fg/µL of genomic DNA extracted from the MTB reference strain H37Rv.	-	100%	[241]
Cas9	Finding Low Abundance Sequences by Hybridization (FLASH)	FLASH to amplify 52 candidate genes probably associated with resistance to first- and second-line drugs in the Mtb reference strain (H37Rv)	Mechanical disruption method	The laboratory H37Rv *Mtb* reference strain, cultured isolates, and sputum	-	-	-	Drug resistance predictions for 15/16 (93.7%) clinical samples	[171]
Cas9	A Cas9/gRNA-assisted quantitative real-time PCR (qRT-PCR) (CARP) assay	S531 and H526 positions in the rifampicin (RIF)-resistance-determining region (RRDR) of the Mtb rpoB gene	-	M. tuberculosis genomic DNA template	~4h	less than 0.0001 femtogram of mycobacterial genomic DNA.	-	100%	[170]
